# Transnasal Ventral Brainstem Epidural Electrical Stimulation Enhances Consciousness in Anesthetized Sheep: A Feasibility Study

**DOI:** 10.1002/cns.70894

**Published:** 2026-04-27

**Authors:** Bin Liu, Jingyu Feng, Xiaofang Zhao, Tao Yu, Zhijun Zhao, Fei Shen, Chenglong Yang, Sihui Wang, Chenyu Nong, Yu Sun, Kaiming Ma, Xin Chen, Guogang Xing, Qinggang Ge, Jun Yang

**Affiliations:** ^1^ Department of Neurosurgery Peking University Third Hospital Beijing China; ^2^ Department of Neurosurgery The First Affiliated Hospital of Baotou Medical College Baotou City China; ^3^ Laboratory Animal Research Center Peking University Third Hospital Beijing China; ^4^ Center for Precision Neurosurgery and Oncology of Peking University Health Science Center Beijing China; ^5^ School of Basic Medical Sciences Peking University Health Sciencze Center and Neuroscience Research Institute Beijing China; ^6^ Department of Intensive Care Medicine Peking University Third Hospital Beijing China

**Keywords:** anesthesia, brainstem stimulation, cortical recording, disorders of consciousness, sheep

## Abstract

**Aim:**

Disorders of consciousness (DOC) remain a major clinical challenge, and the efficacy of currently available neuromodulation strategies remains limited. The brainstem reticular formation is central to arousal regulation, but approaches directly targeting it remain technically demanding. This feasibility study aimed to explore whether epidural electrical stimulation applied via a transnasal clival approach could induce arousal and the electrocorticographic (ECoG) changes.

**Methods:**

Three anesthetized sheep were implanted with epidural stimulating electrodes placed over the ventral brainstem via a transnasal clival approach and cortical recording electrodes on the fronto‐parietal lobes. During the stimulation period, continuous behavioral monitoring was performed, and ECoG was recorded and later processed with spectral analysis.

**Results:**

All three sheep successfully underwent electrode implantation without intraoperative complications, including cerebrospinal fluid leakage or neurovascular injury. Short‐term stimulation reliably elicited behavioral signs of arousal. Concurrently, spectral analysis of ECoG revealed a reduction in low‐frequency power (*δ*, *θ*) and an increase in high‐frequency power (*β*, *γ*).

**Conclusions:**

Transnasal clival epidural stimulation is technically feasible and capable of modulating arousal‐related neural activity. Further investigations with optimized electrode design and larger cohorts are required to validate its safety and efficacy.

## Graphical abstract

This study provides the first experimental evidence of a novel transnasal ventral brainstem epidural electrical stimulation approach in anesthetized sheep and demonstrates that this procedure can induce behavioral arousal and cortical electrophysiological changes toward a wake‐like state, holding promise for the treatment of chronic disorders of consciousness.

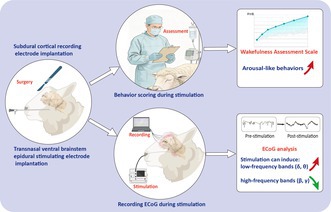

## 1 Introduction

Disorders of consciousness (DOC) represent a spectrum of clinical conditions in which patients lose the normal capacity for awareness of self and environment. They range from coma and the vegetative state/unresponsive wakefulness syndrome to the minimally conscious state [1]. Consciousness is supported by a complex interplay between cortical and subcortical regions, among which the ascending reticular activating system (ARAS) plays an indispensable role [2]. The ARAS originates in the brainstem reticular formation (BRF) and projects widely to the thalamus, basal forebrain, hypothalamus, and neocortex. Through these widespread ascending projections, the ARAS sustains cortical arousal, enables attentional processing, and maintains the neural substrate required for conscious experience [2, 3]. The BRF, a heterogeneous network of neurons distributed across the midbrain, pons, and medulla, lies at the core of consciousness maintenance. Experimental lesion studies and clinical neuroimaging have consistently demonstrated that damage to the BRF or its ascending projections serves as a central mechanism in the pathogenesis of DOC [3, 4].

The current treatment strategies for DOC work by targeting different nodes within the ARAS. Given the limited efficacy of conventional pharmacological treatments, neuromodulation strategies have emerged as promising therapeutic approaches [5, 6]. To date, both invasive and noninvasive techniques have been explored. Deep brain stimulation (DBS) of the central thalamus, particularly the centromedian‐parafascicular (CM‐pf) complex, has been investigated as a therapeutic strategy for DOC patients [7]. Several case reports and small series suggest that thalamic DBS may improve arousal and goal‐directed behavior in a subset of patients, although the response rate remains modest and patient selection criteria are still evolving [7, 8]. Spinal cord stimulation (SCS) has been explored as an alternative neuromodulatory strategy for DOC. Cervical epidural SCS is hypothesized to activate ascending somatosensory and reticulospinal pathways, thereby indirectly engaging the BRF and thalamo‐cortical networks critical for arousal [9, 10]. Clinical studies have reported that approximately 30%–40% of patients demonstrate behavioral or electrophysiological improvements following chronic cervical SCS [9, 11]. Vagus nerve stimulation (VNS), by contrast, is thought to modulate the nucleus tractus solitarius, locus coeruleus, and other components of the BRF through afferent vagal fibers [12]. Preliminary clinical studies and case reports have demonstrated behavioral improvements in a minority of DOC patients following chronic VNS [13]. However, the therapeutic responses of these invasive methods are low to moderate and inconsistent, and the mechanisms remain incompletely understood. In the field of noninvasive stimulation, repetitive transcranial magnetic stimulation (rTMS) applied over the dorsolateral prefrontal cortex has been shown to transiently enhance cortical excitability and network connectivity, with some reports of improved command‐following in DOC patients [14, 15]. Similarly, transcranial direct current stimulation (tDCS) targeting the prefrontal cortex has yielded modest but reproducible gains in behavioral responsiveness, possibly through facilitation of fronto‐parietal networks [16]. Moreover, transcutaneous auricular VNS (taVNS) has also been used to treat DOC, with preliminary data suggesting potential effects on sensorimotor integration and arousal [17]. While encouraging, these noninvasive approaches generally produce only transient improvements, and their efficacy varies widely across individuals.

As seen above, a common feature of current approaches is that stimulation is applied to regions that project indirectly to the ARAS, rather than directly modulating the BRF (the critical hub of ARAS). This raises the question of whether more direct engagement of the BRF could yield stronger and more reliable improvements in consciousness. To address this issue, several animal studies have directly targeted brainstem structures, such as the midbrain tegmentum and the pontine reticular nucleus, and found improvements in consciousness levels [18, 19, 20, 21, 22]. However, the deep location of brainstem nuclei necessitates longer puncture trajectories, increasing procedural risk. Furthermore, the small volume of these nuclei requires high targeting precision, and their dense surrounding structures render direct electrical stimulation prone to adverse effects. These factors have limited the clinical adoption of such puncture approaches.

Given that the BRF comprises a broad network of nuclei and interwoven neural fibers extending throughout the brainstem, stimulation around the brainstem (rather than a precise intraparenchymal point) may more broadly engage the BRF while reducing the risk of damaging discrete brainstem nuclei associated with direct puncture. The cerebellum and temporal lobes shield the dorsal and lateral aspects of the brainstem, whereas its ventral surface offers an alternative access route. From an anatomical standpoint, the nasal route provides easy access to the sphenoid sinus and clivus, and bone removal under navigation guidance allows access to a broad region of the ventral brainstem. Epidural electrical stimulation applied at this site has the theoretical potential to modulate the BRF and restore cortical arousal, thereby offering a novel and potentially more effective neuromodulatory approach for DOC.

Based on these considerations, the present study proposes to develop and evaluate a novel approach of epidural electrical stimulation applied to the ventral surface of the brainstem via a transnasal clival corridor in sheep. By applying epidural electrical stimulation of the BRF at the clivus, this strategy seeks to restore behavioral responsiveness and cortical activity suppressed by isoflurane anesthesia. This line of research is expected to provide proof‐of‐concept evidence for BRF‐targeted neuromodulation and open new therapeutic avenues for patients with chronic DOC.

## 2 Materials and Methods

### 2.1 Animals and MRI Acquisition

**FIGURE 1:**
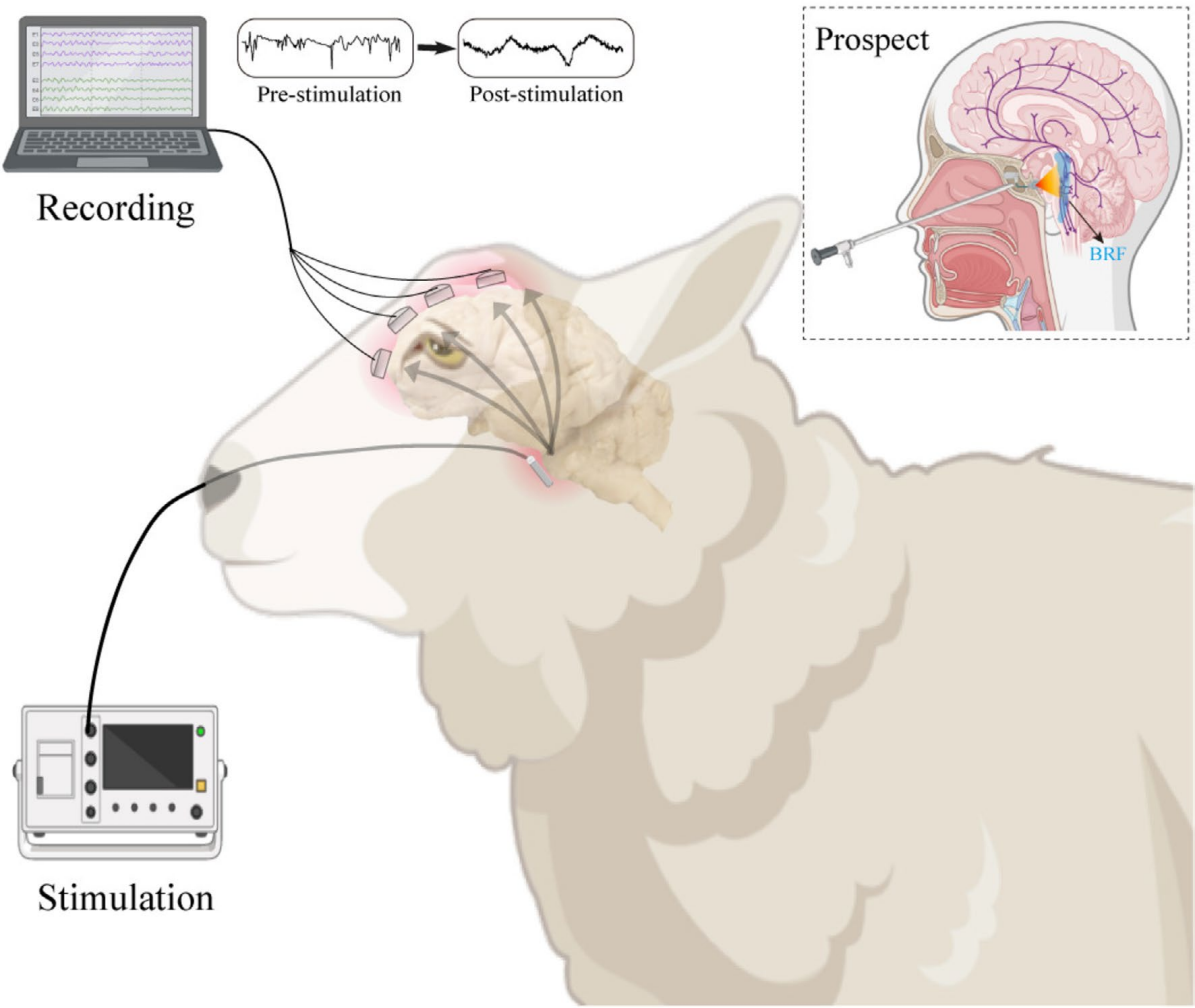
Schematic illustration of the experimental procedure and the clinical application prospect in humans. Through a transnasal endoscopic approach, the clival bone was drilled to expose the epidural space, where a strip stimulation electrode was implanted over the ventral brainstem. Subsequently, subdural recording electrodes were placed over the fronto‐parietal cortex to monitor cortical activity. This technique holds promise for application in patients with disorders of consciousness, in which an optimized epidural electrode could be implanted along the ventral clivus via a transnasal endoscopic approach, while a miniaturized stimulator (capable of wireless Bluetooth charging) could be placed within the sphenoidal sinus to achieve close‐range stimulation of the brainstem reticular formation (BRF).

Three male Chinese Hu sheep (12 months old, 40–50 kg; Beijing Fulong Tengfei Experimental Animal Research Institute) were used in this study. All procedures were approved by the Laboratory Animal Care Management Group of the Institutional Animal Care and Use Committee. Animals were housed at the Experimental Animal Research Center of Peking University Third Hospital for 1 week of preoperative acclimatization before surgery. All surgical procedures were performed under strict adherence to clinical protocols. Experimental design is illustrated in Figure 1.

Structural MRI scans were performed in anesthetized sheep using a 3 T scanner (Prisma, Siemens, Germany) equipped with a 64‐channel head coil to facilitate surgical planning and navigation. Prior to MRI scans, anesthesia was induced by intravenous administration of a mixture of xylazine hydrochloride and Zoletil 50 at a 3:1 ratio (0.3 mL/kg body weight). Endotracheal intubation was performed to maintain airway patency and ensure stable ventilation throughout the procedure. Three‐dimensional T1‐weighted images were acquired using an MPRAGE sequence with the following parameters: TR = 2300 ms, TE = 2.32 ms, flip angle = 8°, slice thickness = 0.9 mm, field of view = 256 × 256 mm, voxel size = 0.9 × 0.9 × 0.9 mm^3^. A three‐dimensional T2‐weighted SPACE dark‐fluid sequence was acquired with the following parameters: TR = 5000 ms, TE = 394 ms, flip angle = 120°, slice thickness = 1.0 mm, field of view = 256 × 240 mm, voxel size = 1.0 × 1.0 × 1.0 mm^3^.

## 3 Surgery

### 3.1 Preoperative Preparation

On the day of surgery, five bone screws were percutaneously implanted into the craniofacial bones of each animal (anesthetized using the same protocol used for MRI acquisition), serving as fiducial markers for image registration. Subsequently, a craniofacial CT scan was performed on each sheep. Subsequently, preoperative MRI and CT images were imported into the NS1 surgical navigation system (Sinovation, Beijing, China) for multimodal image fusion. Following anesthesia and head fixation, the previously implanted bone screws were used for spatial registration to ensure accurate intraoperative localization.

### 3.2 Anesthesia and BIS Monitoring

**FIGURE 2:**
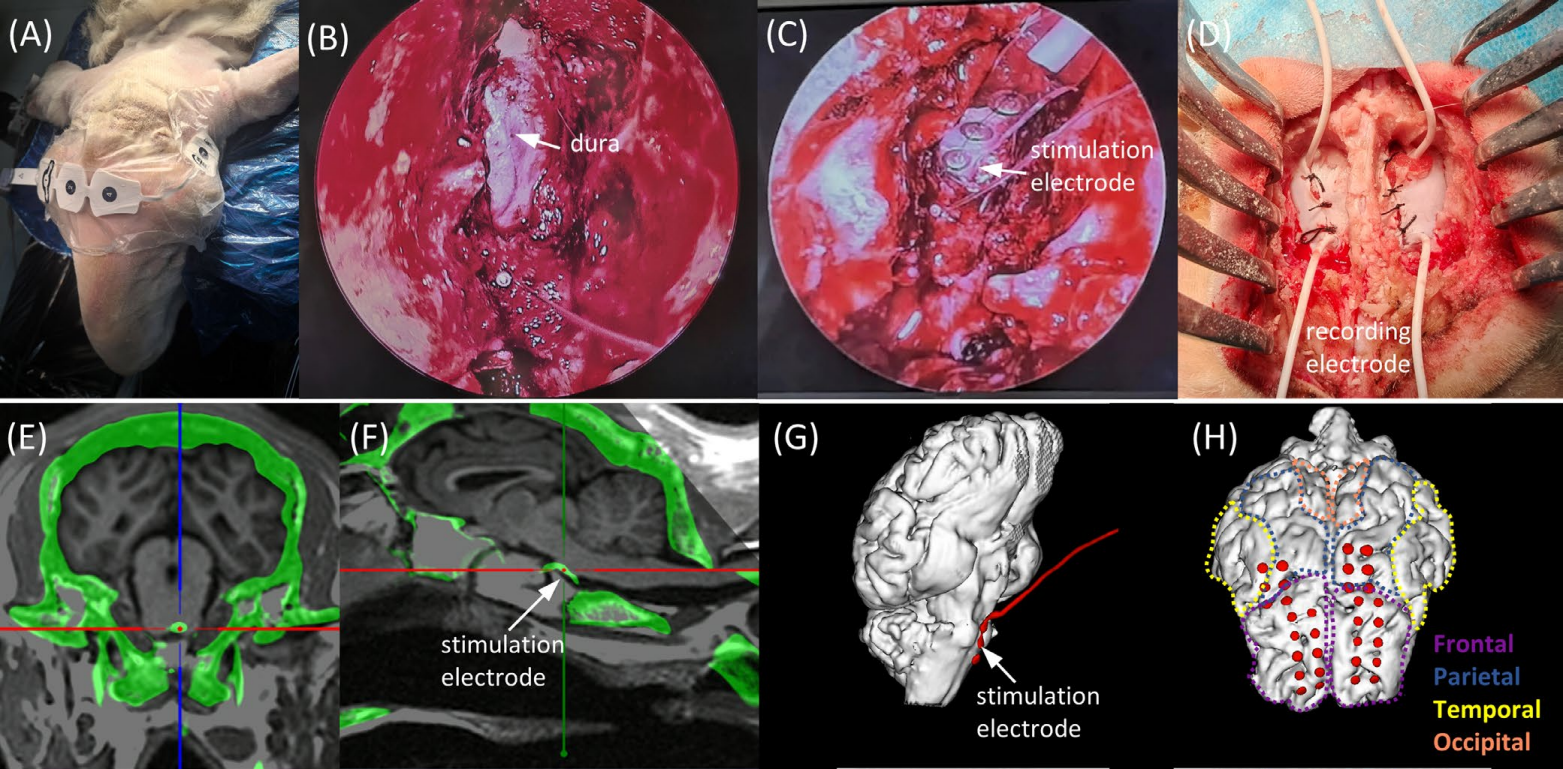
Surgical procedure and postoperative electrode reconstruction. (A) Sheep positioned prone under bispectral index (BIS) monitoring. (B) Endoscopic view showing partial drilling of the clival bone with dura exposure. (C) Epidural implantation of a strip electrode (a total of four contacts) at the ventral brainstem, with the contact 2 (white arrow) responsible for delivering the effective stimulation. (D) Craniotomy over the frontoparietal cortex and implantation of a subdural strip recording electrode. (E) Coronal view of fused CT‐MRI showing the epidural stimulation electrode (cross symbol) anterior to the brainstem. (F) Sagittal view of fused CT‐MRI demonstrating the epidural stimulation electrode at the ventral brainstem. (G) Three‐dimensional reconstruction illustrating the spatial relationship between the stimulation electrode (white arrow) and the brainstem. (H) Three‐dimensional reconstruction showing the spatial distribution of the cortical recording electrodes.

Anesthesia was induced intravenously, after which endotracheal intubation was performed and mechanical ventilation initiated to maintain adequate respiration. An electrocardiographic monitor was connected, and a bispectral index (BIS) sensor was affixed to the cranial surface of each sheep for real‐time monitoring of the depth of anesthesia (Figure 2A). Anesthesia was maintained with inhaled isoflurane at a concentration of 2.3%–2.5% throughout the procedure [23].

### 3.3 Transnasal Ventral Brainstem Epidural Stimulating Electrode Implantation

After anesthesia, the sheep was placed in a prone position with the head immobilized with a head frame. Following successful navigation registration, a nasal endoscope was introduced into the right nasal cavity and advanced along the inferior turbinate to the posterior nasal cavity. Under endoscopic guidance, the mucosa of the posterior nasal septum was incised using an electrocautery hook to create communication between the bilateral posterior nasal cavities. Using a high‐speed drill, the anterior and inferior walls of the sphenoid sinus were removed to expose the clival bone. Stereotactic navigation (Sinovation, Beijing, China) was employed to localize the ventral midbrain and pons, and the thickness of the clival bone at the drilling site was measured for reference. Subsequently, the clival bone was further thinned to create a bone window approximately 1 × 2 cm, exposing the ventral dura mater (Figure 2B). Careful dissection was performed with a dissector to separate adhesions between the dura and clival bone while preserving dural integrity. Under navigation guidance, a strip electrode (diameter 1.5 mm, intercontact space 5 mm, length 20 mm, width 5 mm; manufactured by Sinovation) was inserted into the epidural space (Figure 2C). The electrode was secured with gelatin sponge and fibrin glue. The nasal mucosal flap was repositioned to cover the electrode, and the nasal cavity was gently packed. Electrode leads were fixed and routed transnasally to connect with the neural stimulation generator.

### 3.4 Subdural Cortical Recording Electrode Implantation

The scalp incisions were planned under NS1 navigation guidance. Burr holes were created bilaterally in the frontal regions using a cranial drill, and the bone windows were enlarged with a milling cutter to expose the dura mater. The dura was carefully incised, and two 2 × 8 subdural strip electrodes were placed over the bilateral frontoparietal cortical surfaces (Figure 2D). Electrode leads were secured to the dura with sutures. Bone defects were filled with gelatin sponge, and the scalp incision was sutured. The bilateral cortical recording electrodes were connected to a data acquisition system (NIM‐ECLIPSE, model NS32, Medtronic).

### 3.5 Electrode Verification

An intraoperative CT scan was performed in the anesthetized sheep to verify the placement of both the stimulation and recording electrodes, with adjustments made if the placements were suboptimal. This CT scan was also coregistered with the preoperative MRI for postoperative electrode reconstruction.

### 3.6 Animal Welfare

Sheep underwent a 24‐h fasting period to reduce nausea and vomiting during recovery. Preanesthetic medications (atropine) were administered to reduce respiratory secretions. Anesthesia depth was continuously monitored using a BIS monitor to ensure adequate sedation. Blood pressure, heart rate, and oxygen saturation (SpO_2_) were monitored using a multiparameter monitor to ensure stable physiological parameters throughout the procedure. The operating room temperature was maintained at 24°C–25°C to minimize temperature fluctuations. A surgical blanket was draped over the sheep to cover nonsurgical areas and reduce heat loss. Infused fluids (e.g., sterile saline, anesthetic solution) were warmed to 37°C using a fluid warmer to prevent hypothermia. Core body temperature was noninvasively monitored using an ear thermometer at 15‐min intervals throughout the experiment. After the experiment, to minimize unnecessary suffering, all the sheep were euthanized by a trained veterinary technician via an overdose of pentobarbital sodium (5% w/v; 150 mg/kg body weight) intravenously administered at 5–10 mL/min.

## 4 Stimulation and Data Collection

### 4.1 Adjustment of Anesthesia

The isoflurane concentration was reduced from 2.3%–2.5% to 1.0%–1.5%, resulting in an increase in BIS values from 40–50 to 60–70, which was maintained for at least 10 min to allow the depth of anesthesia to restabilize before neural activity recording.

### 4.2 Stimulation and Cortical Recording

A total of 10 repeated epidural stimulations were performed. For each trial, the electrocorticographic (ECoG) activity was recorded for 90 s before stimulation (baseline), followed by 30 s of electrical stimulation (5 mA, 100us, 100 Hz), and a 2‐min poststimulation recording. This design ensured an approximate 4‐min interval between individual stimulations, minimizing potential carry‐over effects from the previous stimulation.

### 4.3 Behavior Observation

One dedicated evaluator who was blinded to the study hypothesis scored the arousal behaviors of the sheep using an adapted animal wakefulness assessment scale [24]. Six behavioral items were assessed: exploration of the surrounding environment (0: absence; 1: small search of external clue; 2: total investigation of the environment), spontaneous movements (0: absence; 1: small torso and/or limb movement; 2: large torso and/or limb movement), shaking/prodding (0: nothing; 1: small body movement; 2: large body movement), toe pinch (0: nothing; 1: body movement or eye blinking or cardiac rate change; 2: body movement and eye blinking or eye opening and cardiac rate change), eye opening (0: nothing; 1: small blinks or eye movements; 2: full eye opening), and corneal reflex (0: absent; 1: present). To avoid interference with poststimulation ECoG recordings, behavioral assessments were conducted exclusively during the 30‐s stimulation period (10 times).

## 5 Cortical ECoG Data Processing

### 5.1 Preprocessing

Data preprocessing was implemented using the EEGLAB toolbox in MATLAB R2024b [25]. Initially, transient spike detection and repair were performed by calculating a dynamic threshold (three times the standard deviation) for each channel. Signal segments exceeding this threshold were identified as artifacts and corrected via linear interpolation (interp1) to mitigate electrode transients or amplifier saturation effects. Subsequently, a 6th‐order Butterworth low‐pass filter (250 Hz cutoff) with zero‐phase bidirectional filtering (filtfilt) was applied to eliminate high‐frequency noise while preserving phase integrity, followed by a 3rd‐order Butterworth band‐stop filter (48–52 Hz stopband) for 50 Hz powerline interference suppression. To address nonstationary pulse noise induced by electrical stimulation, sliding median filtering (15‐point window) with mirror‐padded boundaries was employed to minimize edge artifacts. Finally, bipolar derivation referencing was implemented across all recording sites to attenuate common reference electrode contamination and enhance the spatial specificity of localized signal variations.

### 5.2 Event‐Related Spectral Perturbation (ERSP) Analysis

Continuous cortical ECoG recordings were epoched relative to stimulation onset such that time zero (0 s) corresponds to the start of stimulation. For each trial, a −30 s to +150 s window was extracted, covering the prestimulation baseline (−30 s–0 s), stimulation period (0 s–30 s), and poststimulation recording (30 s–150 s). Trials with gross artifacts were rejected before spectral analysis (one, two, and zero trials were excluded for each sheep, respectively).

The absolute time‐frequency power for each trial was derived by the short‐time Fourier transform (STFT), which employs a Hanning window (2 s duration) with 75% window overlap to minimize interframe information loss. The number of fast Fourier transform (FFT) points was set to the nearest integer power of 2 relative to the window length, ensuring consistent frequency binning across the analysis. This analysis covered a frequency range of 0 to 50 Hz to capture the dynamics of major neural oscillatory rhythms.

To quantify stimulus‐induced relative changes in power, we conducted the ERSP analysis. First, the mean power at each frequency, *P*_*baseline*_(*f*), was calculated across the baseline period (−30s to 0 s). Then, the power value at each time‐frequency point, *P*(*t*, *f*), was converted to a decibel (dB) scale relative to the baseline mean power using the following formula. The results were averaged across all trials to obtain the average time‐frequency map for each cortical recording contact.

$$\text{ERSP}{\left(t,f\right)}_{\left(\mathrm{dB}\right)}=10*\log_{10}\left(\frac{\;P\left(t,f\right)}{P_{\text{baseline}}\left(f\right)}\right)$$

To summarize the overall response pattern across all contacts and frequency bands, the ERSP values were averaged within a poststimulus effect window (30 s–60 s, to avoid artifacts caused by stimulation) and across predefined frequency bands: *δ* (0–4 Hz), low *θ* (4–6 Hz), high *θ* (6–9 Hz), *α* (9–14 Hz), *β* (14–35 Hz), low *γ* (35–55 Hz), medium *γ* (55–85 Hz), and high *γ* (85–125 Hz) [26].

### 5.3 Statistical Analysis

To compare the power differences across each frequency band before and after stimulation, we conducted trial‐level statistical analyses for each sheep—specifically, we extracted the mean ERSP values of each trial and subsequently performed one‐sample *t*‐tests. Considering that the positions of cortical contacts varied across individual sheep, yet the first five rows of contacts were localized to the frontal lobe and the last three rows to the parietal lobe in all three sheep, we classified all cortical monitoring contacts into four anatomical groups (the left frontal lobe, right frontal lobe, left parietal lobe, and right parietal lobe) for statistical analyses. A *p*‐value < 0.05 was set as the statistical significance.

## 6 Results

### 6.1 Transnasal Ventral Brainstem Electrical Stimulation Is Technically Feasible

Under the guidance of neuroendoscopy and neuronavigation, epidural electrode implantation at the clivus was successfully achieved in all three sheep. The ventral brainstem dura was well exposed without cerebrospinal fluid leakage or vascular injury. Postoperative three‐dimensional reconstruction confirmed satisfactory placement of the epidural electrode at the clivus and appropriate positioning of the cortical electrodes (Figure 2E–H), with the anterior five rows of contacts covering the frontal lobe surface and the posterior three rows covering the parietal lobe surface.

### 6.2 Electrical Stimulation of the Ventral Brainstem Can Induce Arousal Behavior

**FIGURE 3:**
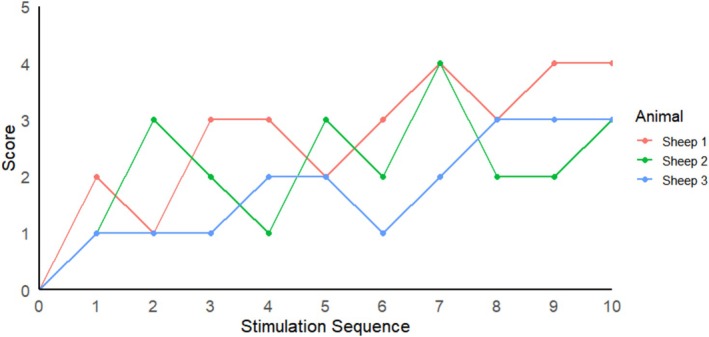
Changes in arousal behavior scores following epidural ventral brainstem stimulation in three sheep. The *x*‐axis represents the sequence of repeated stimulation trials, and the *y*‐axis represents the arousal behavior scale scores.

Following epidural brainstem stimulation, the sheep exhibited increased scores on the wakefulness assessment scale. Across 10 repeated stimulations (120 s intervals), the scores showed an upward trend, indicating a cumulative effect (Figure 3). These increases were primarily attributable to spontaneous movements of the trunk and limbs, as well as motor responses and eye blinking elicited by toe pinching.

### 6.3 Electrical Stimulation of the Ventral Brainstem Can Induce Changes in Cortical Spectral Power

**FIGURE 4:**
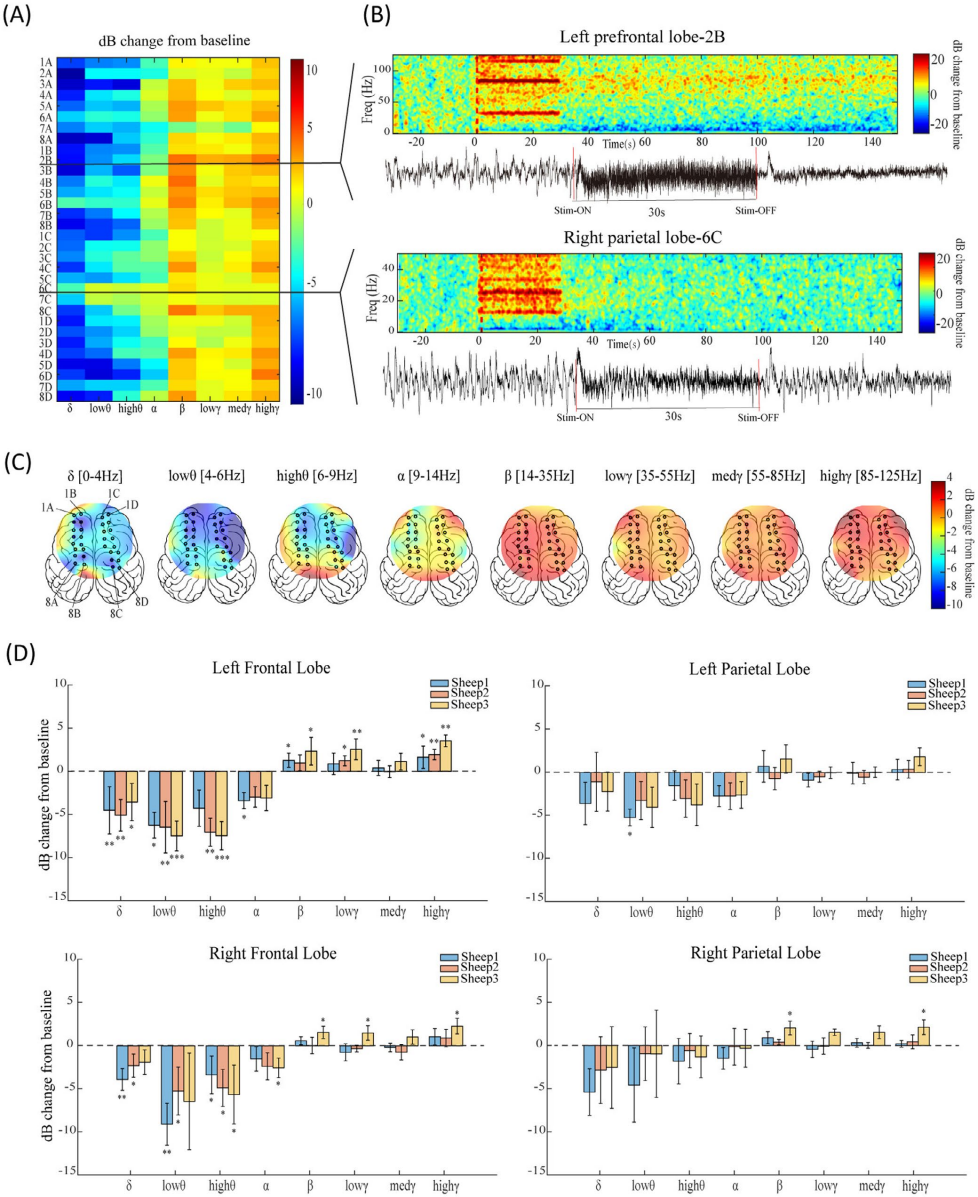
Cortical spectral power changes induced by epidural ventral brainstem stimulation. (A) Power spectrum alterations across all cortical recording channels before and after stimulation. Blue indicates poststimulation power decreases, while red indicates poststimulation power increases. Each row corresponds to one electrode channel. Channels 1A‐8B are located in the left hemisphere and 1C‐8D in the right hemisphere, arranged from frontal to parietal regions with increasing numbers. (B) Representative time‐frequency plots and raw EEG signals from channels 2B and 6C. It reveals a dynamic redistribution of spectral power following the 30‐s epidural stimulation, characterized by a pronounced suppression of low‐frequency oscillations and a concurrent enhancement of high‐frequency power, especially for the frontal contact (2B). The color scale represents the intensity of the power (dB) changes relative to the baseline mean. The red dashed line denotes the onset of stimulation (time 0), which lasted for 30 s. (C) Topographical maps of cortical spectral power changes across different frequency bands following epidural electrical stimulation. Black dots denote the positions of cortical monitoring electrode contacts. (D) The relative changes in cortical spectral power of different frequency bands induced by stimulation of each sheep, stratified by four anatomical brain regions: The left frontal lobe, left parietal lobe, right frontal lobe, and right parietal lobe. The downward bars represent a power decrease after stimulation, while the upward bars indicate a power increase. *p < 0.05, **p < 0.01, ***p < 0.001.

We investigated the spatial and temporal changes across different frequency bands. The ERSP results confirmed a robust pattern of power decrease in low‐frequency bands (*δ*, *θ*, *α*) and a marked power increase in high‐frequency bands (*β*, *γ*) when comparing the poststimulation period to the prestimulation baseline (Figure 4A,B). This stimulus‐induced power modulation was bilateral and widespread, with the most prominent effects observed in the bilateral frontal lobes, where the power in the low‐frequency bands (*δ*, *θ*, *α*) decreased and the power in the high‐frequency bands (*β*, *γ*) increased (Figure 4C,D).

## 7 Discussion

In this study, we developed and validated a novel transnasal approach for epidural stimulation of the ventral brainstem in an anesthetized sheep model. We demonstrated that repetitive stimulation via this route successfully elicited measurable behavioral arousal, accompanied by cortical electrophysiological changes toward a wake‐like state. These findings provide the first experimental evidence that epidural stimulation of the ventral brainstem delivered through a transnasal route can directly modulate brainstem‐cortical arousal circuits.

The choice of an appropriate animal model is critical for the translational investigation of such a novel stimulation paradigm. Sheep provide an attractive large‐animal model for several reasons. First, the sheep brain is gyrencephalic and closer to the human brain in terms of size, anatomy, and white matter composition compared to rodents [27]. This makes sheep particularly suitable for testing neurosurgical procedures and electrode implantation strategies that require sufficient cranial volume and realistic anatomical landmarks. Second, the clival anatomy of sheep allows transnasal access to the ventral brainstem, providing a feasible platform for developing and validating epidural stimulation techniques [28]. Although the nasal passages in sheep are deeper and the angle between the nasal corridor and clivus is steeper than in humans, these differences can be compensated for by extending surgical instruments and adjusting the working angle. Third, sheep are generally docile and easy to handle, which reduces stress‐related confounds during experimental procedures. All these features make sheep a practical and relevant large‐animal model for investigating brainstem stimulation and its effects on cortical and behavioral arousal.

General anesthesia provides a reversible and controllable model for probing the mechanisms of DOC. Among anesthetic agents, isoflurane is particularly relevant because its neural effects recapitulate several core features observed in chronic DOC [29, 30]. Isoflurane acts primarily by enhancing GABAergic inhibition and suppressing excitatory glutamatergic transmission, leading to widespread cortical and thalamocortical deactivation [31]. Importantly, studies have shown that isoflurane disrupts the ARAS and functional connectivity across frontoparietal networks, both of which are essential for sustaining wakefulness and awareness [32]. Moreover, research has shown that cerebral functional connectivity exhibits dose‐specific spatiotemporal alterations with varying isoflurane concentrations. At 1.0%–1.5% isoflurane (the concentration we used during epidural electrical stimulation and cortical recording), the functional connectivity could remain stable, and the cortex remains responsive to electrical stimulation [33, 34]. Thus, although anesthesia is pharmacologically induced and differs from brain injury‐related DOC in etiology, the underlying neurophysiological mechanisms share important commonalities, making anesthetic models a practical and ethically acceptable framework for probing mechanisms of unconsciousness and testing potential neuromodulatory interventions [32].

The transnasal clival approach offers a direct and anatomically rational pathway to the ventral surface of the brainstem. By carefully removing clival bone and applying epidural stimulation, this route enables close‐range modulation of the BRF. More broadly, the nasal cavity provides a potential gateway to multiple deep brain regions (from front to back: the orbitofrontal cortex, basal forebrain, pituitary, hypothalamus, thalamus, and brainstem), embodying the emerging “From Nose to Brain” paradigm in neuromodulation [35]. Previous studies have shown that stimulation of the olfactory mucosa can alter deep functional connectivity [36], and Guo et al. [37] recently introduced the “DeepFocus” minimally invasive approach, demonstrating that transnasal stimulation through the cribriform plate and sphenoidal sinus can access reward‐related circuits at the skull base. Building upon this concept, our study extends the application toward brainstem modulation through the clival corridor, further underscoring the translational potential of transnasal approaches for targeted neuromodulation. In future applications, miniaturized electrodes and battery systems could be implanted within the sphenoidal sinus, fully leveraging its anatomical cavity to enable wireless stimulation while avoiding the risks associated with intracranial implants (Figure 1). Collectively, these insights highlight the promise of transnasal stimulation as a versatile and scalable strategy for brain‐machine interfacing and neuromodulation.

Despite its promise, transnasal clival stimulation must carefully address the safety concerns associated with this anatomical region. The clivus lies in proximity to critical neurovascular structures, including the basilar artery and multiple cranial nerves, whose injury could lead to devastating neurological consequences. Nevertheless, recent advances in neurosurgical technology, such as three‐dimensional neuroendoscopy, neuronavigation, surgical robotics, navigation‐integrated drill, and intraoperative ultrasound, substantially enhance the precision of transnasal procedures and reduce the risk of complications. Specifically, to ensure procedural safety, we recommend performing preoperative high‐resolution MRI and angiographic scanning. These imaging datasets will then be coregistered with preoperative localizing CT scans in the surgical navigation system, allowing us to individually determine the optimal bone resection sites to avoid the anatomical course of the basilar artery. Intraoperatively, the target bone resection sites will be identified under direct visualization with a neuroendoscope. A navigation‐integrated drill—with a navigation reference frame affixed to the drill handle—will be used for cautious bone ablation, which enables real‐time monitoring of the distance between the drill tip and the clival dura mater. Once the bone is sufficiently thinned, a laminectomy rongeur will be employed to enlarge the bone window. Of note, the lack of histological verification and chronic data creates a gap between animal experiments and clinical translation in humans. In the future, chronic data will be needed to further verify its stability and safety. For instance, it is necessary to clarify the mucosal healing situation, as well as the risk of infection, electrode displacement, chronic fibrosis or inflammation, etc.

In the present study, we observed that epidural stimulation of the ventral brainstem induced obvious behavioral arousal and ECoG transition in anesthetized sheep. These findings align with previous animal studies that directly target the brainstem. For instance, Muindi et al. [19] demonstrated that electrical stimulation of the parabrachial nucleus in isoflurane‐anesthetized rats resulted in behavioral arousal and restoration of the righting reflex, accompanied by a significant decrease in cortical *δ* power. Similarly, Pillay et al. [20, 21] found that stimulation of the pontine nucleus oralis during light anesthesia resulted in cortical desynchronization, characterized by a decrease in *δ*‐ and *θ*‐band power. Additionally, Vincent et al. [18] reported that electrical stimulation of the ventral tegmental area generates broadband decreases in power for frequencies < 20 Hz reflected by frontal cortical recordings in anesthetized rats. These studies collectively demonstrate that stimulation of various brainstem structures can elicit cortical electrophysiological changes similar to those observed in our experiments. This indicates that our epidural stimulation likely exerted its effects by modulating the BRF, which in turn induces the observed alterations in cortical activity.

This study has several limitations. First, the sample size was small, which limited the subject‐level statistical analyses. However, as a feasibility study, these preliminary findings provide a proof of concept, and future studies will require larger cohorts to validate and generalize the results. Second, the stimulation parameters were based on previous epidural stimulation protocols [38, 39], but the optimal parameters for ventral brainstem epidural stimulation remain to be systematically explored. Third, the behavioral scoring system includes subjective components. Video recording, along with independent scoring by multiple blinded evaluators, is needed in future work to further mitigate bias. Fourth, this study did not include control groups, making it difficult to eliminate the interference of some nonspecific factors (e.g., lightening of anesthesia). Nevertheless, we used a within‐animal, multitrial, prepost comparison design to calculate power changes, which could provide a relatively robust assessment of stimulus‐specific effects. Future experiments, including sham, off‐target, or subthreshold stimulations, are warranted to enhance the rigor of the study. Finally, this study only provided a foundational step for restoring arousal (physiological state of wakefulness) and did not address whether the stimulation improved awareness (conscious content). This was mainly attributed to the acute nature of our experimental design (with immediate euthanasia following stimulation trials), which precluded the implementation of prolonged, task‐based behavioral paradigms required to indirectly assess awareness in nonhuman animals. Future investigations in chronic models enabling long‐term behavioral monitoring and cognitive assays for awareness will be necessary to explore the potential effects of this stimulation strategy on higher‐order conscious functions.

## 8 Conclusion

In this study, we introduced a novel neuromodulation approach targeting the ventral brainstem via a transnasal epidural route to improve wakefulness of anesthetized sheep, supporting its potential as a feasible intervention for DOC. However, the procedure carries inherent risks due to the proximity of critical neurovascular structures, underscoring the need for advanced intraoperative guidance technologies and optimized electrode designs. This is a proof‐of‐concept feasibility study, and future studies with larger sample sizes and long‐term assessments are warranted to validate both the efficacy and safety of this approach.

## Author Contributions

Study conception and design: J.Y., Q.G., and T.Y.; data collection: B.L., J.F. and X.Z.; analysis and interpretation of results: J.F., B.L., C.Y., S.W., C.N., F.S. and Z.Z.; writing – original draft preparation: B.L., J.F. and Y.S.; writing – review and editing: K.M., X.C. and C.Y.; supervision: J.Y., G.X. and Q.G.; funding acquisition: J.Y. and B.L. All authors have read and agreed to the published version of the manuscript.

## Funding

This work was supported by the Young Scientists Fund of National Natural Science Foundation of China (No. 82501756), the China Postdoctoral Science Foundation (No. 2025M772125), and the National Natural Science Foundation of China (No. 82272675).

## Disclosure

The authors have nothing to report.

## Ethics Statement

The experimental protocol conformed to national guidelines for animal usage in research and was approved by the Ethics Committee of Peking University Third Hospital (No. IRB00006761‐A20250035).

## Conflicts of Interest

The authors declare no conflicts of interest.

## Data Availability

The data that support the findings of this study are available from the corresponding author upon reasonable request.
